# Effect of gel seat cushion on chronic low back pain in occupational drivers: A double-blind randomized controlled trial

**DOI:** 10.1097/MD.0000000000012598

**Published:** 2018-10-05

**Authors:** Jang Woo Lee, Young-Ho Lim, Yu Hui Won, Dong Hyun Kim

**Affiliations:** a Department of Physical Medicine and Rehabilitation, National Health Insurance Service Ilsan Hospital, Goyang; b Department of Physical Medicine and Rehabilitation, Hallym University Sacred Heart Hospital, Hallym University College of Medicine, Anyang; c Department of Physical Medicine and Rehabilitation, Chonbuk National University Medical School, Jeonju; d Department of Physical Medicine and Rehabilitation, Kangdong Sacred Heart Hospital, Hallym University College of Medicine, Seoul, Korea.

**Keywords:** gel cushion, low back pain, occupational driver

## Abstract

**Background:**

Low back pain (LBP) is an exceedingly common medical condition that results in significant medical and social burden. Sitting for a long period is a common aggravating factor for LBP. Although seat cushion is known to promote comfort, relieve pressure, and correct posture, its effect on chronic LBP has not yet been investigated. This study aimed to evaluate the clinical effect of gel seat cushion on chronic LBP in occupational drivers.

**Methods:**

Occupational drivers with chronic LBP lasting for >6 months were recruited. Subjects were double-blinded, randomly assigned to 2 groups (gel and foam cushion groups), and instructed to use the provided cushions while driving. Pain threshold and tissue hardness were measured at tender points using a digital algometer. Numeric pain intensity scale (NPIS), Roland–Morris Disability Questionnaire (RMDQ), and Oswestry Disability Index (ODI) were used to analyze the primary endpoint, whereas the Beck Depression Inventory and Short Form-6D were used for the secondary endpoint.

**Results:**

Of 80 enrolled subjects, 75 (gel cushion group, 40; foam cushion group, 35) were included for analysis. Both groups showed significant improvement in NPIS and ODI scores following cushion use. Results for Beck Depression Inventory and Short Form-6D scores indicated that gel cushion use was significantly helpful. Change in NPIS score was significantly greater in the gel cushion group than in the foam cushion group.

**Conclusion:**

Gel cushion use may be effective in relieving LBP in occupational drivers seated for a long period compared with foam cushion use.

## 1 Introduction

Low back pain (LBP) is an exceedingly common medical condition with a lifetime prevalence rate of 84%.^[1]^ Known pain generators include the nerves, bones, musculatures, fascia, joints, and ligaments; however, nonphysical factors such as educational, occupational, or psychosocial status are also closely related to pain generation.^[2,3]^ A multidisciplinary approach that includes medical treatment and lifestyle modifications (e.g., exercise and posture correction) should be implemented to effectively treat LBP.^[2,4]^

LBP is prone to recurrence and, in certain cases, may develop into chronic LBP, which is defined as persisting pain lasting for more than 3 months.^[5]^ In severe cases, LBP may result in some form of functional disability, interfering with patients’ occupational activities, which may in turn impose individual and social burden.^[6,7]^

Various biomechanics are associated with LBP development, with an individual's posture and daily activities being particularly closely related to it.^[8]^ Maintaining a seated position for a long period is of particular importance as a common aggravating factor for LBP.^[9,10]^ A seat cushion is known to promote comfort, relieve pressure, and correct posture.^[11–14]^ However, its effect on chronic LBP has not yet been investigated. This study aimed to evaluate the clinical effect of gel seat cushion on chronic LBP in occupational drivers who spent a relatively long period in a seated position and in a certain specific posture.

## 2 Methods

### 2.1 Cushions

**Figure 1:**
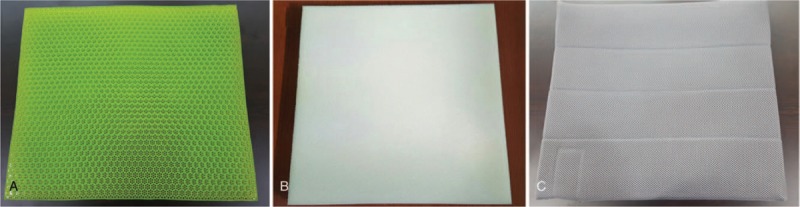
Gel and foam cushions evaluated in this study. (A) Uncovered gel cushion, (B) uncovered foam cushion, and (C) covered cushion.

In this study, a gel cushion (Balanceseat, Bullsone Co., Seoul, Korea) manufactured with thermoplastic styrene–ethylene/butylene–styrene elastomers was used. The cushion, with a size of 42 × 40 × 2.5 cm, comprised numerous small hexagonal cells that distribute pressure, mitigate any physical impact, and promote air permeability. To evaluate the effect of the gel cushion, we prepared a plain rectangular polyurethane foam cushion that had the same size as the gel cushion. Each cushion was placed in a cover of similar design, which had a bottom made of nonslippery materials (Fig. 1).

### 2.2 Subjects

Occupational drivers with chronic LBP lasting for >6 months who drove >6 hours per day were recruited; those aged 40 to 65 years with a score of >4 on the numeric pain intensity scale (NPIS) at the initial evaluation were considered candidates. Occupational drivers who underwent previous lumbar surgery or invasive lumbar interventions within the preceding 3 months or had definite traumatic etiology, as well as those with: signs of dural irritation, weakness in the lower extremity, and scoliosis with Cobb angle of >20° that were detected through detailed physical examinations were excluded from the study. Subjects were double-blinded and randomly assigned to 2 groups; they were instructed to use the provided seat cushion while driving for 3 months and were notified in advance that arbitrarily discontinuing cushion use or undergoing any surgical or invasive procedure for back pain would disqualify them from the study.

### 2.3 Design of trial

Sample size was calculated by G power. With assumption that significant difference of NPIS is 1.0 and standard deviation is 1.5, the effect size was calculated as 0.667. Calculated sample size of each group was 37. And we planned to recruit 90 subjects for this research expecting 20% of them would be dropped out. Screening questionnaire including pain severity, painful area, and medical history was distributed to occupational drivers who participated in regular conservative education program performed by the government institution. We selected possible candidates based on the questionnaire and confirmed the subjects by the telephone survey. Selected subjects were instructed to visit hospital for research participation at their available time. Each subject was allocated to one of 2 groups by drawing lots. Researcher who assigned the subjects to study groups did not intervene the study process after assignment of allocation. Care provider and statistical processor were all blinded from the group of each subject.

### 2.4 Outcome measurement

We randomly distributed one of 2 cushions to all subjects and instructed them to use it while driving. The primary endpoint was NPIS score, which was evaluated using a self-rating questionnaire. A digital algometer (OE-220, ITO, Tokyo, Japan) was used to measure pain threshold (kg/cm^2^) and tissue hardness (N/cm^2^) at tender points. With respect to subjects’ functional status, the Korean versions of the Roland–Morris Disability Questionnaire and the Oswestry Disability Index (ODI) were used to analyze the primary endpoint. In relation to the secondary endpoint, the Beck Depression Inventory and Short Form-6D were used to measure psychological outcome and quality of life, respectively. All questionnaires concerning functional status were completed on a self-rating basis. Subjects visited the clinic prior to the start of the study and at 3 months after study entry for questionnaire completion and measurement of pain threshold and tissue hardness, which was performed by a single rehabilitation specialist blinded to cushion types being used. A picture of the evaluated area with markers was taken during initial measurement of pain threshold and tissue hardness so that subsequent evaluations at the same area could be performed. At 1 month after study entry, evaluations of the status of cushion use were performed via telephone interviews.

Paired *t* test was used for the analysis of the measured before and after use of each cushion. And for comparison between gel and form cushions, analysis of covariance (ANCOVA) was used with values of before study and treatment groups as covariance using SPSS version 22 (IBM, Armonk, NY).

This study was approved by the institutional review board of Kangdong Sacred Heart Hospital, Hallym University College of Medicine (2015-06-011).

## 3 Results

**Figure 2:**
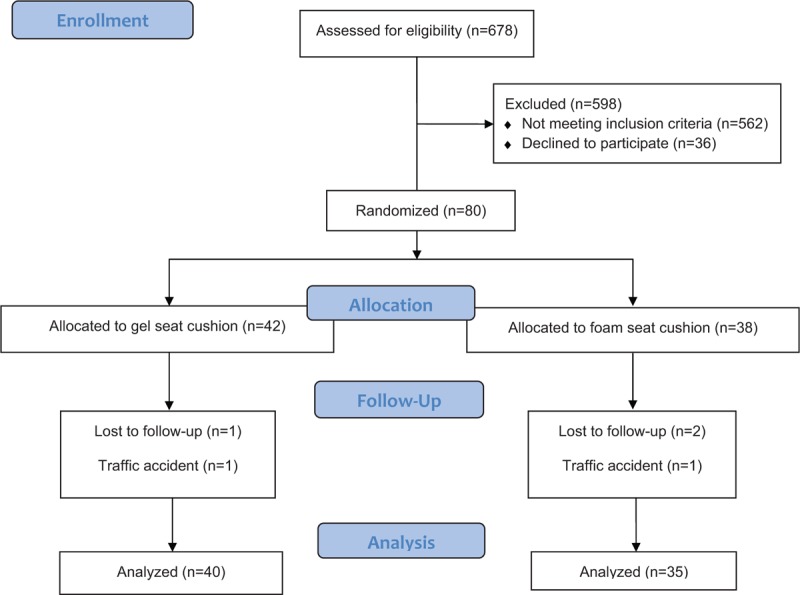
Flow of participation.

Among 90 selected subjects, a total of 80 subjects visited the hospital. They were randomly allocated into one of the 2 groups (gel cushion group, 42; foam cushion group, 38) (Fig. 2). At the time of enrollment, sex restriction was not imposed to possible candidates, but all selected subjects turned out to be male. In the gel cushion group, 2 subjects were lost during the study, 1 was lost to follow-up, and the other was excluded owing to a traffic accident. In contrast in the foam cushion group, 3 subjects were lost, 2 were lost to follow-up, and 1 was excluded because of a traffic accident. Overall, 75 subjects (gel cushion group, 40; foam cushion group, 35) were included for analysis. Traffic accidents were not related to cushion use in any way, and no subject discontinued cushion use owing to discomfort or underwent any surgical and/or invasive procedure. At the time of enrollment, no statistically significant differences (including in age) were observed among subjects in both groups (Table 1).

**Table 1:**
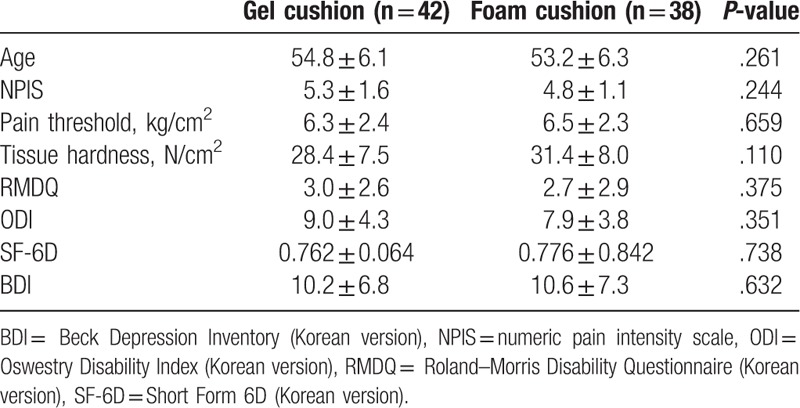
Demographic characteristics of the each study group.

Subjects in the gel cushion group reported statistically significant improvement in NPIS, ODI, and Short Form-6D scores and in pain threshold after using the gel cushion. In contrast, subjects in the foam cushion group exhibited statistically significant improvement in NPIS and ODI scores only. Change in NPIS score in the gel cushion group was significantly greater than in the foam cushion group (Table 2).

**Table 2:**
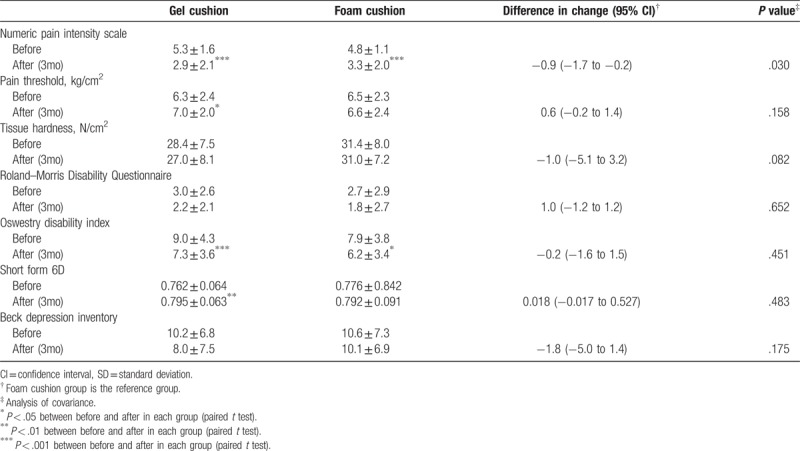
Mean (±SD) outcome values before and after use of each cushions, and difference in changes from before study.

## 4 Discussion

Occupational drivers are constantly exposed to the risk of back pain owing to the seated position that they are required to maintain for a long period and the continuous vibration caused by motor vehicle driving. This study excluded factors such as physical modalities, manipulative intervention, and surgical or invasive procedure and detected clinical improvement in chronic LBP solely through cushion use. Cushion use is not popular among drivers in South Korea but could be a very effective method for back pain management in occupational drivers, considering that it is simpler, more cost-effective, less time-consuming, and less prone to adverse effects than other medical approaches to back pain treatment.

In our view, the clinical improvement in chronic LBP associated with gel cushion use in our study could be attributed to the following mechanisms. First, the improvement could be attributed to the effect of posture correction. A seat cushion's positive effect on sitting posture has been reported in a previous biomechanical study.^[13,14]^ A seat cushion is very commonly used by wheelchair-bound patients with paralysis to prevent physical deformities such as scoliosis and promote musculoskeletal system stability. Further, in light of a previous study that concluded that seat cushion use could lead to posture correction even in healthy individuals,^[13]^ posture correction induced by gel cushion use possibly contributed to the improvement in LBP in this study as well.

Second, vibration due to motor vehicle driving has been reported to principally induce strain in the lumbar spine.^[15]^ The improvement in LBP was possibly induced via shock absorption by gel elastomers in the cushion used in the study, blocking the transmission of motor vehicle vibration to the subjects’ body.

Third, musculature relaxation may also have contributed to the improvement in LBP. Measurement of pain threshold using a pressure algometer is a commonly used method to evaluate pain of muscular origin. The correlation between the increase in an individual's pain threshold and the decrease in pain has been reported in another study.^[16,17]^ Further, the reduction in muscle hardness, which refers to muscle stiffness, is closely related to muscle relaxation and pain reduction.^[17–19]^ In this study, there was no significant change in muscle hardness; however, a change in pain threshold was observed in the gel cushion group, although a significant difference between groups was not observed using ANCOVA. It might be inferred that the use of the gel cushion resulted in relaxation of the musculature.

In an automobile, occupant seat pressure is known as a major determinant of postural comfort.^[20]^ Moreover, seat pressure relief is related to the improvement in blood circulation.^[21]^ In this study, pressure mapping was not performed; however, a previous study on pressure mapping has shown that a gel cushion was more efficient in relieving pressure than a foam cushion.^[22,23]^ As such, we believe that the gel cushion used in the study was more effective in distributing pressure than the foam cushion and that this may have led to the improvement in blood circulation and pain reduction.

This study has limitations. First, the explanation on the relationship between gel cushion use and our study findings was insufficient owing to the absence of anthropometric and biomechanical evaluation. Second, evaluation or pressure mapping to confirm whether posture correction was achieved through gel cushion use was not performed in this study. We believe that a further laboratory study addressing this would be required. Third, despite detailed physical examinations and radiography, the enrolled group was relatively heterogeneous with respect to the causes of back pain owing to a lack of further diagnostic evaluation such as magnetic resonance imaging and electromyography. It should also be noted that the study duration was relatively short compared with the duration of relevant pain.

## Acknowledgments

The authors thank Bullsone Co., Ltd. for the support.

## Author contributions

**Conceptualization:** Jang Woo Lee.

**Data curation:** Jang Woo Lee.

**Formal analysis:** Jang Woo Lee.

**Investigation:** Jang Woo Lee, Young-Ho Lim.

**Methodology:** Jang Woo Lee.

**Project administration:** Dong Hyun Kim.

**Supervision:** Dong Hyun Kim.

**Validation:** Yu Hui Won.

**Writing – original draft:** Jang Woo Lee, Young-Ho Lim, Yu Hui Won, Dong Hyun Kim.

**Writing – review & editing:** Dong Hyun Kim.

Dong Hyun Kim orcid: 0000-0002-3102-385X
